# How I do it: flexible endoscopic aspiration of intraventricular hemorrhage

**DOI:** 10.1007/s00701-020-04499-z

**Published:** 2020-07-23

**Authors:** Alberto Feletti, Luca Basaldella, Alessandro Fiorindi

**Affiliations:** 1 Department of Neurosciences, Biomedicine and Movement Sciences, Institute of Neurosurgery, University of Verona, AOUI Verona, Polo Chirurgico “P. Confortini”, P.le Stefani 1, 37126 Verona, Italy; 2 Unit of Neurosurgery, Treviso Regional Hospital, Treviso, Italy; 3 Unit of Neurosurgery, Spedali Civili, University of Brescia, Brescia, Italy

**Keywords:** IVH, Hemorrhage, Endoscopy, Flexible, Brain ventricles, EVD, Aneurysm

## Abstract

**Background:**

As intraventricular blood is a strong negative prognostic factor, intraventricular hemorrhage requires prompt and aggressive management to reduce intracranial hypertension.

**Method:**

A flexible scope can be used to navigate and to aspirate blood clots from all four ventricles. Complete restoration of CSF pathways from the lateral ventricle to the foramen of Magendie can be obtained.

**Conclusion:**

Flexible neuroendoscopic aspiration of IVH offers the opportunity to immediately reduce intracranial hypertension, reduce EVD obstruction and replacement rates, and decrease infections and shunt dependency.

**Electronic supplementary material:**

The online version of this article (10.1007/s00701-020-04499-z) contains supplementary material, which is available to authorized users.

## Relevant surgical anatomy

During the endoscopic aspiration of intraventricular hemorrhage (IVH), the vision of anatomical landmarks is severely impaired. A detailed knowledge of intraventricular anatomy is therefore mandatory before facing this type of surgery [2, 5, 7, 8].

## Description of the technique

**Fig. 1:**
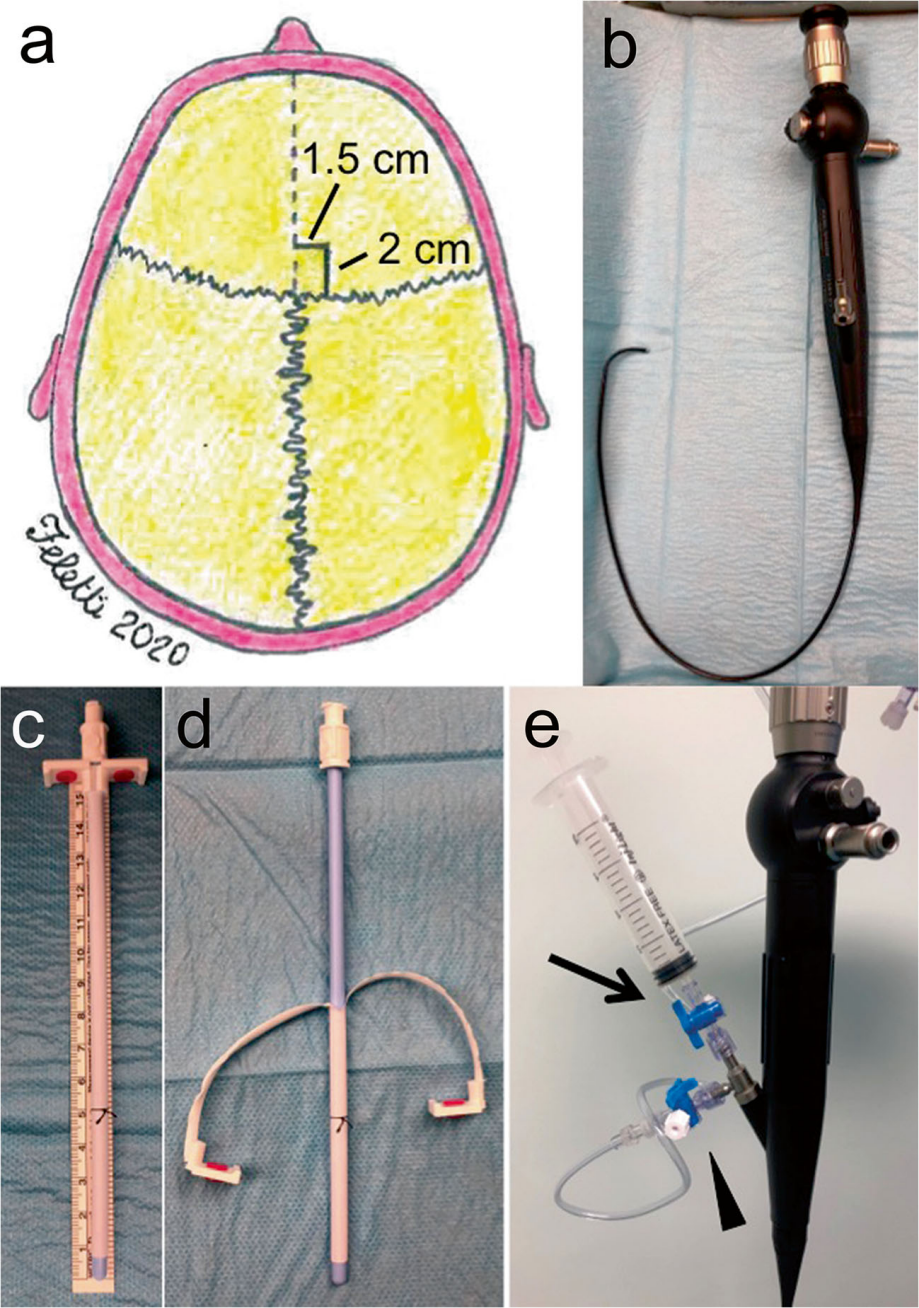
**a** The precoronal burr hole should be placed at about 1.5 cm from the midline. **b** Flexible endoscope (Karl Storz, Tuttlingen, Germany). **c** The peel-away length is marked using a stitch placed at about 5 cm from the tip. **d** After cannulation of the ventricle, the peel-away is opened and the mandrel removed. **e** A 20-ml syringe (arrow) and a catheter (arrowhead) are connected to the outer ends of the scope for aspiration and irrigation, respectively

**Fig. 2:**
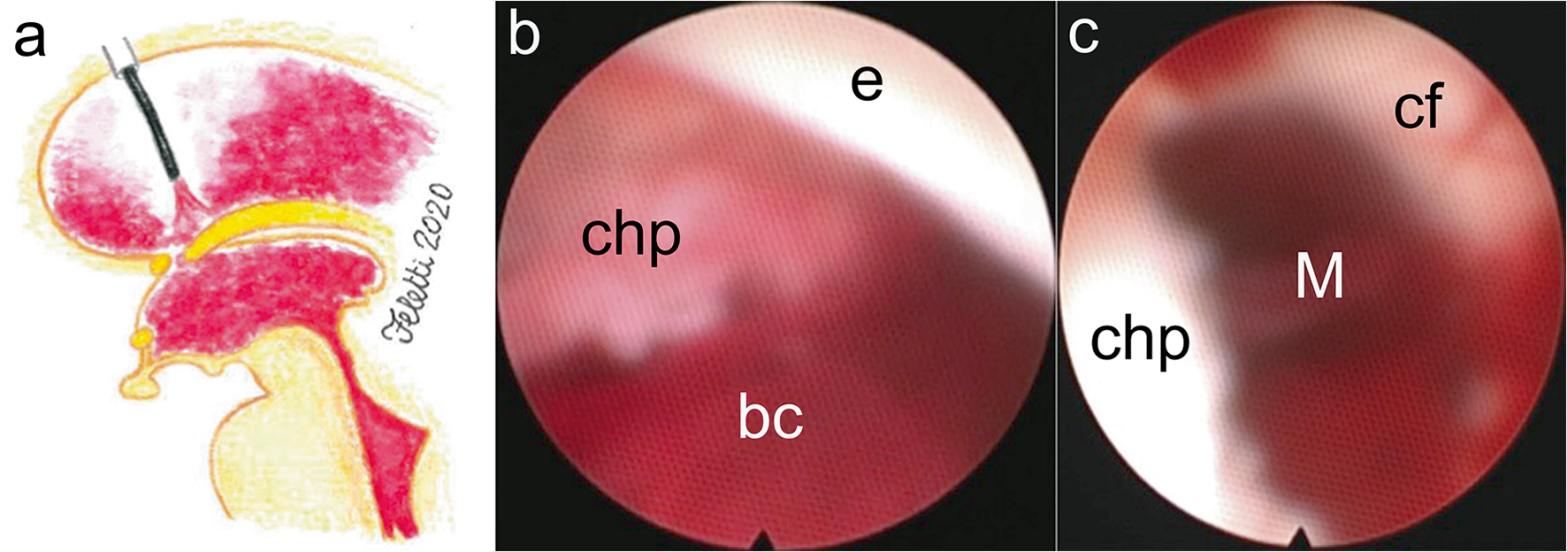
Clots aspiration from the lateral ventricle. **a** Artist’s illustration showing the tip of the endoscope in the lateral ventricle. **b** The tip of the scope is directed backwards: after the initial aspiration of blood clots (bc), the choroid plexus (chp) and the ependyma (e) of the floor and lateral wall of the right lateral ventricle are unveiled. **c** Directing the tip of the scope anteriorly, the choroid plexus (chp) leads to the foramen of Monro (M), contoured by the column of the fornix (cf) and still obstructed by clots

A precoronal, paramedian 3-cm-long, curved skin incision is performed at the side of the lateral ventricle with the largest amount of blood. The burr hole must be placed not too laterally, at about 1.5 cm from the midline (Fig. 1). This is crucial in order to have a more straight direction of the scope towards the cerebral aqueduct and to be able to smoothly enter the fourth ventricle later on during the procedure. Lateral ventricle is cannulated with a semirigid 14-French peel-away introducer catheter, through which the flexible endoscope is inserted (Fig. 1). Different types of flexible scopes are available in the market with external diameter ranging from 2.8 to 5 mm. However, external diameter should not be larger than 4 mm for this kind of procedure (Fig. 1). Such size can fit the diameter of the cerebral aqueduct allowing its safe navigation. The diameter of the operative channel ranges from 1.2 to 1.5 mm. When the lateral ventricle is explored, the screen appears completely red or dark because the tip of the scope is dipped in blood. Although the severely impaired vision might be worrisome and frustrating, intermittent aspirations and irrigation using an external 20-ml syringe connected to the outer end of the scope allow the breaking and the initial removal of the clots. The second operator adjusts the suction force depending on the resistance he can perceive directly on his hands. In any case, the aspirations must be stopped immediately after seeing whitish color on the screen, because this means that the ependymal layer has been unveiled already, and they must be alternated with Ringer lactate irrigations in order to clear the field and improve the vision. In this way, the operative channel of the scope is used as a sucker and an irrigator. The choroid plexus is usually the first anatomical landmark to be unveiled and must be followed backwards to remove as much blood as possible from the occipital horn and forwards until the foramen of Monro is identified (Fig. 2). In order to remove blood clots from the contralateral ventricle, although contralateral approach through a different burr hole has been described, a standard septostomy can be performed.

**Fig. 3:**
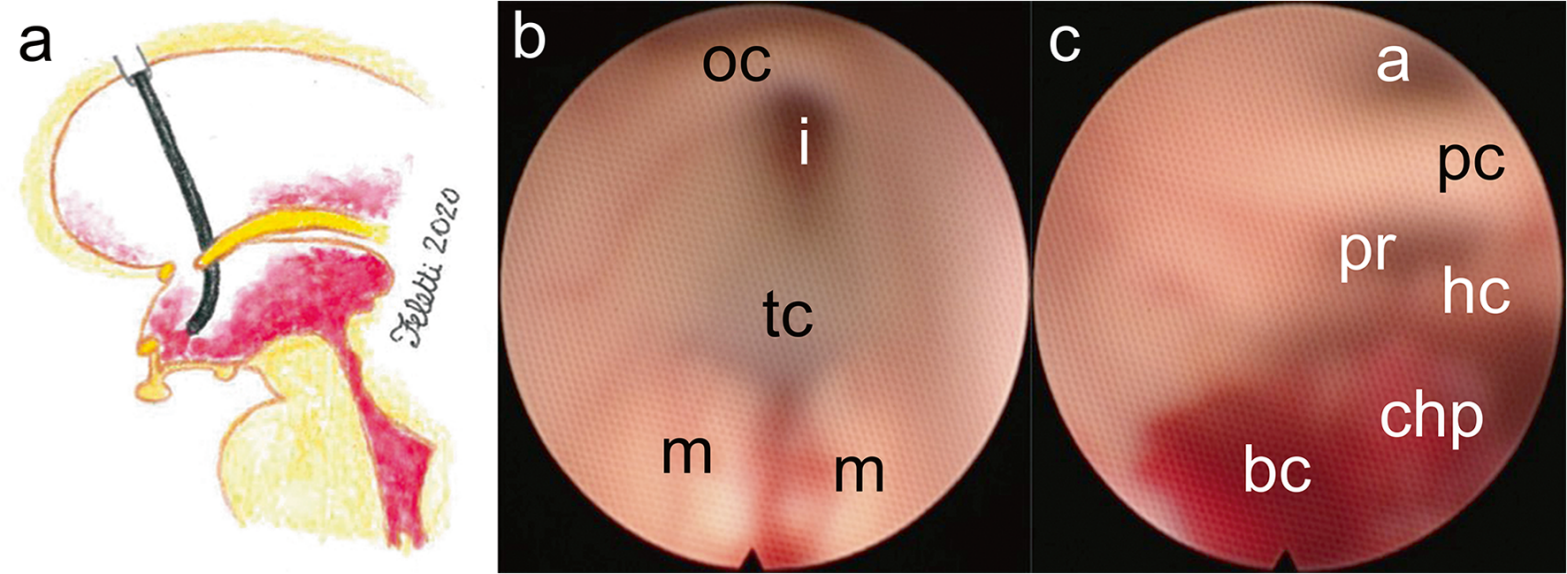
Clots aspiration from the third ventricle. **a** Artist’s illustration showing the direction of the endoscope through the foramen of Monro into the third ventricle. **b** Appearance of the anterior third ventricle after clots aspiration: mammillary bodies (m) and *tuber cinereum* (tc) are usually the first anatomical landmarks that can be seen, followed by the *infundibulum* (i) and the optic chiasm (oc). **c** Residual blood clots (bc) in the posterior third ventricle. The choroid plexus on the roof of the third ventricle, the habenular commissure (hc), the pineal recess (pr), the posterior commissure (pc), and the adytum of the cerebral aqueduct (a) are visible

**Fig. 4:**
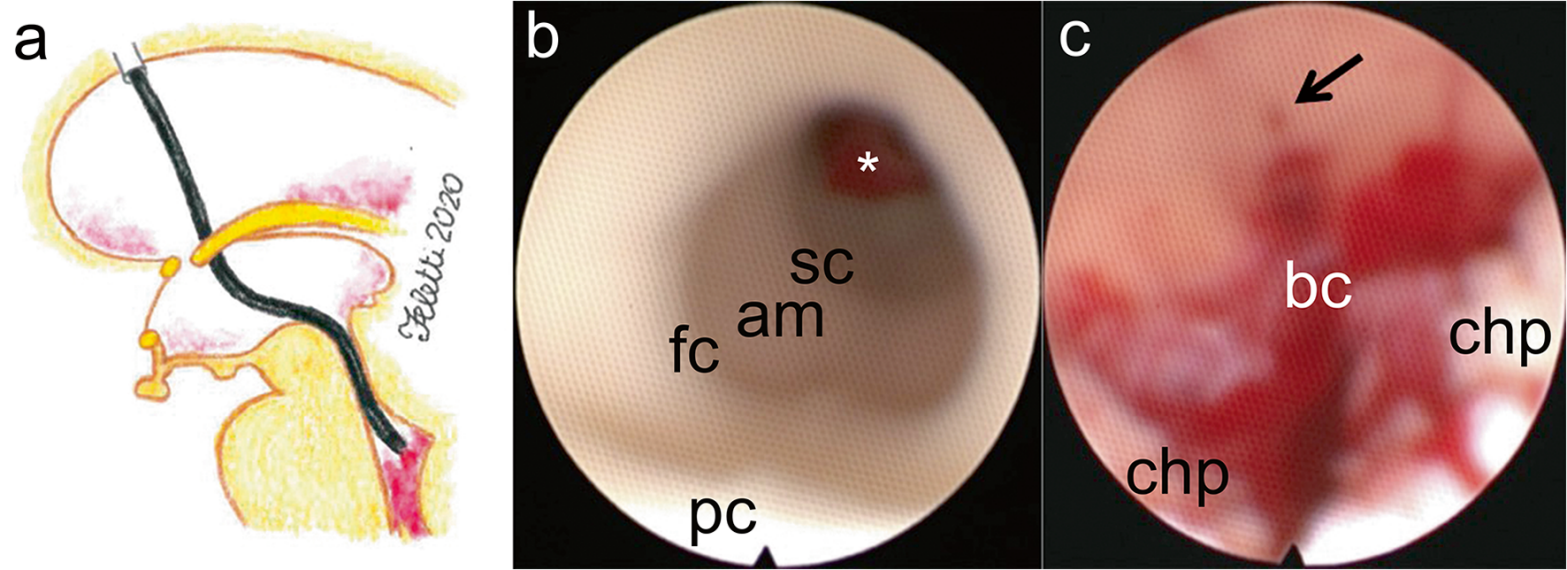
Clots aspiration from the fourth ventricle. **a** Artist’s illustration showing the transaqueductal navigation of the fourth ventricle. **b** Closer inspection of the cerebral aqueduct reveals the first constriction, the ampulla (am), the second constriction (sc), and blood clot hampering the *egressus* of the cerebral aqueduct (*). **c** The inferior triangle of the fourth ventricle: after blood aspiration, the choroid plexus (chp) on the roof of the ventricle and the *canalis centralis medullaris* at the *calamus scriptorius* (black arrow) are now visible. Blood clots (bc) are still obstructing the foramen of Magendie

**Fig. 5:**
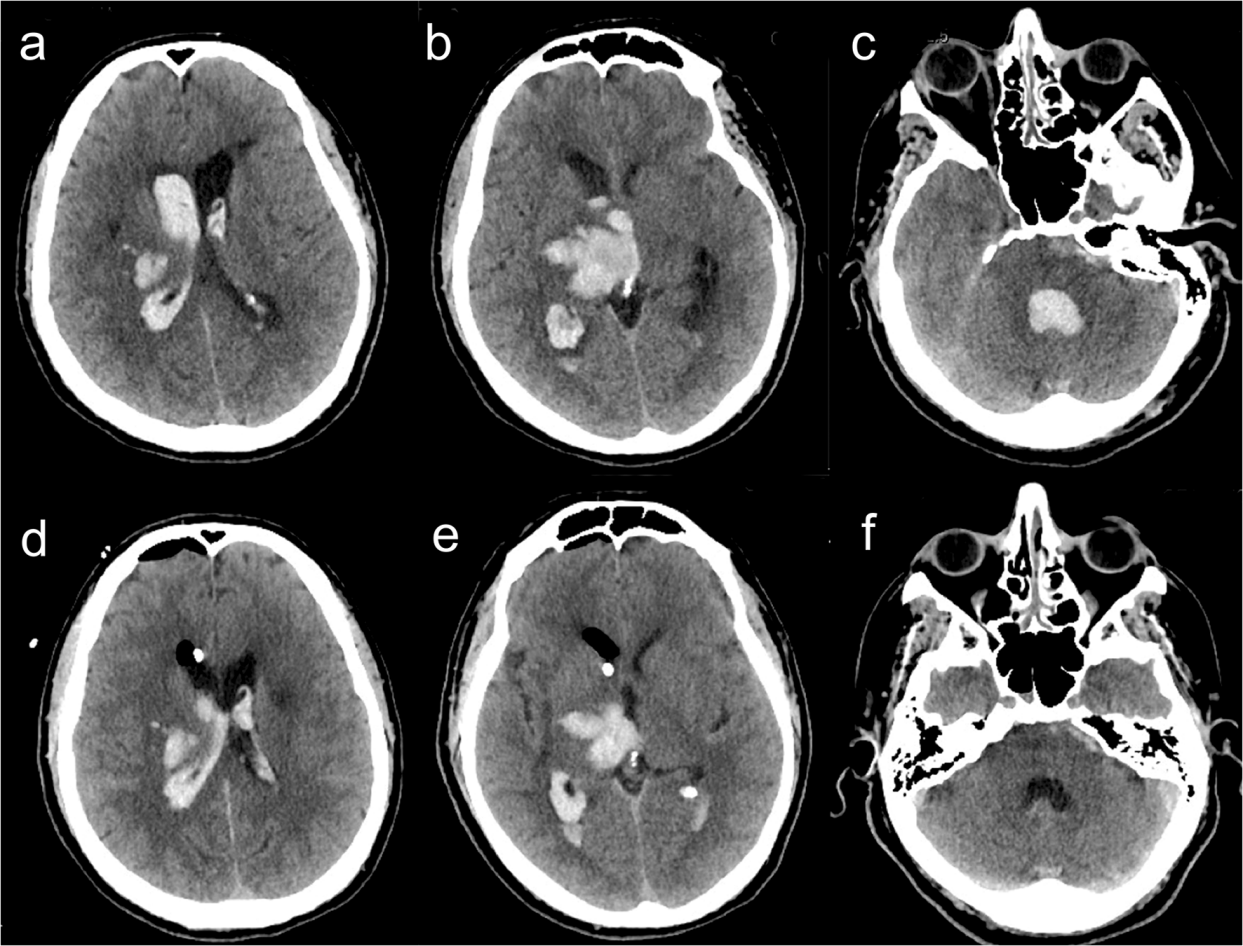
**a**, **b**, **c** Preop CT scan showing a thalamic hemorrhage with intraventricular extension. **d**, **e**, **f** Immediate postop CT scan after endoscopic IVH aspiration

**Fig. 6:**
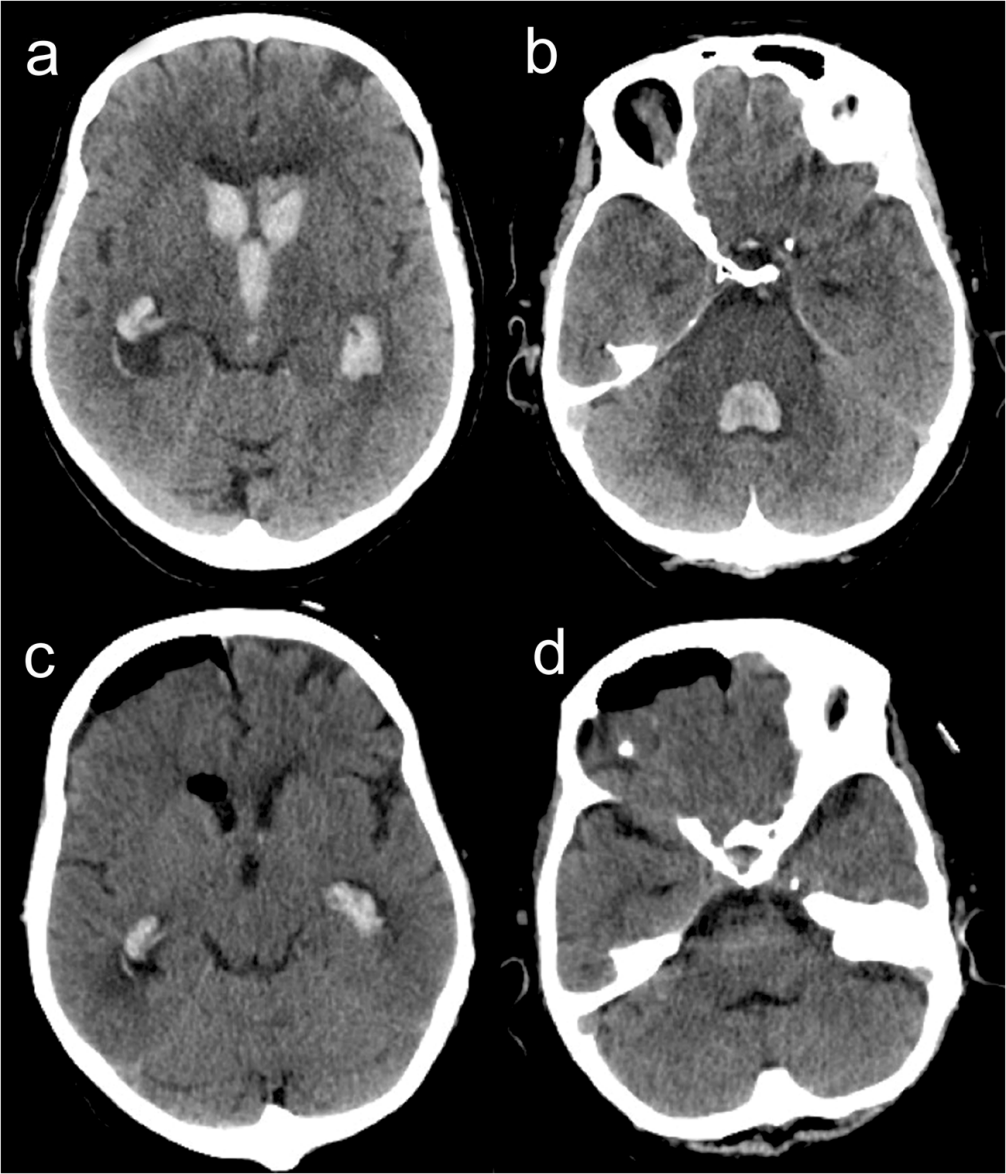
**a**, **b** Preop CT scan showing a tetraventricular hemorrhage in a patient affected by Moyamoya disease. **c**, **d** Immediate postop CT scan after endoscopic IVH aspiration

The scope is advanced into the third ventricle, where further aspirations and irrigations expose the mammillary bodies and *tuber cinereum*. The tip of the scope is then bent towards the posterior part of the third ventricle, where the posterior and habenular commissures along with the adytum of the cerebral aqueduct can be seen (Fig. 3). The clots removal from the fourth ventricle finally resolves the obstruction at the level of the foramina of Luschka and Magendie (Fig. 4). It is crucial to set the third and the fourth ventricles free of blood in order to restore the patency of the whole CSF pathway (Figs. 5 and 6).

At the end of the procedure, after ruling out any active source of bleeding, an EVD is left in place for both ICP monitoring and CSF drainage [4].

## Indications

Any patient presenting with IVH and requiring an EVD because of intracerebral hypertension or obstructive hydrocephalus. In case IVH is due to aneurysm or AVM rupture, the vascular malformation is secured at first [3].

## Limitations

1. Quality of vision is currently conditioned by the presence of optical fibers.
2. If surgery is not performed within 48–72 h from the bleeding, clots might be already organized and harder to break and aspirate.
3. IVH aspiration of blood clots is not a single-surgeon procedure: an assistant surgeon is always required.

## How to avoid complications

1. Place the burr hole not too lateral in order to have a smooth access through the cerebral aqueduct.
2. Promptly stop aspiration as soon as the whitish color of the ventricular wall is seen.
3. Carefully balance aspiration and irrigation volumes, especially in the fourth ventricle. As the endoscope occupies the entire diameter of the cerebral aqueduct hampering CSF or Ringer’s solution outflow, excessive irrigation into the fourth ventricle can determine local hypertension and bradycardia.
4. Prolonged direct irrigation is sufficient to stop eventual bleeding. Sometimes, gently pushing an inflated Fogarty balloon against the bleeding source can speed up the bleeding control by compression.
5. When withdrawing the endoscope from the fourth and the third ventricles, care must be taken to exactly retrace the same route used previously, in order to avoid damages to the ependymal walls, veins, and choroid plexus.

## Specific perioperative considerations (pre- and postop workup, instructions for the postop care)

Rule out coagulation or aggregation disorders before surgery or correct coagulation diathesis. Administer prophylactic anticonvulsants.

Make sure that Ringer lactate has been warmed up at body temperature preoperatively.

Mean arterial pressure should be kept below 110 mmHg and perfusion pressure greater than 70 mmHg.

Carefully calculate the depth of the peel-away before insertion.

Inspect with the endoscope through the peel-away, and open it only after confirming that its inferior edge is positioned at the level of the ependyma of the roof of the lateral ventricle.

CT scan is performed immediately after surgery. Keep EVD open at about 15 cm from the tragus, and try to wean it off during the following 2–3 days.

It is necessary to master simpler procedures with flexible scope as septostomy or third ventriculostomy, before attempting endoscopic removal of IVH.

## Specific information to give to the patient about surgery and potential risks

The endoscopic aspiration of IVH is performed in an urgent setting, as the patient is unconscious. However, the relatives must be made aware of both advantages and risks of the procedure. Among the advantages are quick restoration of CSF pathways, decrease of intracranial hypertension, reduction of EVD obstruction and replacement rates, consequent reduction of infections, and potential decrease of shunt dependency [1, 10]. Moreover, the amount of intraventricular blood is a strong prognostic factor, and its fast removal has been associated with better outcome [6, 9].

Among the risks are iatrogenic hemorrhage due to peel-away insertion or inadvertent damage to intraventricular vessels, basal ganglia infarction due to vein tearing at the foramen of Monro, and diplopia in case of damage of the periaqueductal gray matter, cardiac arrhythmias, and infections.

## Electronic supplementary material

Video 1 IVH aspiration from the lateral ventricle and the anterior part of the third ventricle. (MP4 40,476 kb)

Video 2 IVH aspiration from the posterior part of the third ventricle, the cerebral aqueduct, and the fourth ventricle. (MP4 54,704 kb)
